# Vaccination timing of low-birth-weight infants in rural Ghana: a population-based, prospective cohort study

**DOI:** 10.2471/BLT.15.159699

**Published:** 2016-06-01

**Authors:** Maureen O’Leary, Sara Thomas, Lisa Hurt, Sian Floyd, Caitlin Shannon, Sam Newton, Gyan Thomas, Seeba Amenga-Etego, Charlotte Tawiah-Agyemang, Lu Gram, Chris Hurt, Rajiv Bahl, Seth Owusu-Agyei, Betty Kirkwood, Karen Edmond

**Affiliations:** a Department of Infectious Disease Epidemiology, Faculty of Epidemiology and Population Health, London School of Hygiene & Tropical Medicine, Keppel St, London, WC1E 7HT, England.; b Institute of Primary Care and Public Health, University of Cardiff, Cardiff, Wales.; c Engender Health, New York, United States of America (USA).; d Department of Community Health, Kwame Nkrumah University of Science and Technology, Kumasi, Ghana.; e Kintampo Health Research Centre, Kintampo, Ghana.; f Institute of Cancer and Genetics, University of Cardiff, Cardiff, Wales.; g Department of Maternal, Newborn, Child and Adolescent Health, World Health Organization, Geneva, Switzerland.; h School of Paediatrics and Child Health, University of Western Australia, Crawley, Australia.

## Abstract

**Objective:**

To investigate delays in first and third dose diphtheria–tetanus–pertussis (DTP1 and DTP3) vaccination in low-birth-weight infants in Ghana, and the associated determinants.

**Methods:**

We used data from a large, population-based vitamin A trial in 2010–2013, with 22 955 enrolled infants. We measured vaccination rate and maternal and infant characteristics and compared three categories of low-birth-weight infants (2.0–2.4 kg; 1.5–1.9 kg; and < 1.5 kg) with infants weighing ≥ 2.5 kg. Poisson regression was used to calculate vaccination rate ratios for DTP1 at 10, 14 and 18 weeks after birth, and for DTP3 at 18, 22 and 24 weeks (equivalent to 1, 2 and 3 months after the respective vaccination due dates of 6 and 14 weeks).

**Findings:**

Compared with non-low-birth-weight infants (*n* = 18 979), those with low birth weight (*n* = 3382) had an almost 40% lower DTP1 vaccination rate at age 10 weeks (adjusted rate ratio, aRR: 0.58; 95% confidence interval, CI: 0.43–0.77) and at age 18 weeks (aRR: 0.63; 95% CI: 0.50–0.80). Infants weighing 1.5–1.9 kg (*n* = 386) had vaccination rates approximately 25% lower than infants weighing ≥ 2.5 kg at these time points. Similar results were observed for DTP3. Lower maternal age, educational attainment and longer distance to the nearest health facility were associated with lower DTP1 and DTP3 vaccination rates.

**Conclusion:**

Low-birth-weight infants are a high-risk group for delayed vaccination in Ghana. Efforts to improve the vaccination of these infants are warranted, alongside further research to understand the reasons for the delays.

## Résumé

**Objectif:**

**Examiner les retards d'administration de la première et de la troisième dose de vaccin diphtérie–tétanos–coqueluche (DTP1 et DTP3) chez les nourrissons au faible poids de naissance au Ghana, ainsi que les déterminants associés.**

**Méthodes:**

**Nous avons utilisé les données issues d'un vaste essai sur la vitamine A, mené dans la population en 2010–2013, et qui portait sur 22 955 nourrissons. Nous avons déterminé le taux de vaccination ainsi que les caractéristiques des mères et des enfants et avons comparé trois catégories de nourrissons au faible poids de naissance (2,0–2,4 kg; 1,5–1,9 kg; et < 1,5 kg) avec des nourrissons pesant **≥** 2,5 kg. Une régression de Poisson nous a permis de calculer les ratios des taux de vaccination pour le DTP1 à 10, 14 et 18 semaines après la naissance et, pour le DTP3, à 18, 22 et 24 semaines (ce qui équivaut respectivement à 1, 2 et 3 mois après l'âge normal de vaccination qui est de 6 et 14 semaines).**

**Résultats:**

**Comparés aux nourrissons n'ayant pas un faible poids de naissance (*n* = 18 979), ceux au faible poids de naissance (*n* = 3382) avaient un taux de vaccination DTP1 presque 40% plus faible à l'âge de 10 semaines (ratio des taux ajusté, RTa: 0,58; IC 95%: 0,43–0,77) et à l'âge de 18 semaines (RTa: 0,63; IC 95%: 0,50-0,80). Les nourrissons pesant de 1,5 à 1,9 kg (*n* = 386) avaient un taux de vaccination à ces âges environ 25% plus faible que ceux pesant **≥** 2,5 kg. Des résultats similaires ont été observés pour le DTP3. Le plus jeune âge des mères, leur niveau d'instruction et les distances plus longues jusqu'à l'établissement de soins le plus proche étaient associés à de plus faibles taux de vaccination DTP1 et DTP3.**

**Conclusion:**

**Les nourrissons au faible poids de naissance sont un groupe à haut risque en matière de retard de vaccination au Ghana. Des efforts devraient être entrepris pour améliorer la vaccination de ces enfants, parallèlement à d'autres recherches permettant de comprendre les raisons de ce retard.**

## Resumen

**Objetivo:**

Investigar los retrasos de la primera y tercera dosis de la vacuna contra la difteria, el tétanos y la tos ferina (DTP1 y DTP3) en recién nacidos con insuficiencia ponderal en Ghana, así como los determinantes relacionados con las mismas.

**Métodos:**

En 2010–2013, se utilizaron datos de un ensayo poblacional de vitamina A a gran escala basado en la población con 22 955 recién nacidos inscritos. Se midió la tasa de vacunación y las características tanto de las madres como de los recién nacidos, y se compararon tres categorías de recién nacidos con insuficiencia ponderal (2,0–2,4 kg; 1,5–1,9 kg; y < 1,5 kg) con recién nacidos con un peso de ≥ 2,5 kg. Se utilizaron modelos de regresión de Poisson para calcular los coeficientes de la tasa de vacunación para DTP1 las semanas 10, 14 y 18 después del nacimiento, y para DTP3 las semanas 18, 22 y 24 (lo que equivale a 1, 2 y 3 meses tras las fechas de vencimiento de las vacunaciones correspondiente de las semanas 6 y 14).

**Resultados:**

En comparación con los recién nacidos sin insuficiencia ponderal (*n* = 18 979), los que nacieron con bajo peso (*n* = 3 382) tenían una tasa de inmunización sistemática de DTP1 casi un 40% inferior a la edad de 10 semanas (razón de tasa ajustada, aRR: 0,58; IC del 95%: 0,43–0,77) y a la edad de 18 semanas (aRR: 0,63; IC del 95%: 0,50–0,80). Los recién nacidos con un peso de 1,5–1,9 kg (*n* = 386) tenían unas tasas de vacunación de alrededor de un 25% inferior a los que pesaban ≥ 2,5 kg a la misma edad. Para DTP3 se observaron los mismos resultados. Se asociaron la juventud maternal, el bajo nivel educativo y la larga distancia hasta la instalación sanitaria más cercana con las bajas tasas de vacunación de DTP1 y DTP3.

**Conclusión:**

Los recién nacidos con insuficiencia ponderal se encuentran en un grupo de alto riesgo para sufrir un retraso de la vacunación en Ghana. Se han garantizado esfuerzos para mejorar la vacunación de estos recién nacidos, junto con una investigación más profunda para comprender las razones de dichos retrasos.

## ملخص

**الغرض:**

استقصاء التأخر في تلقي الجرعة الأولى والثالثة من اللقاح ضد الدفتريا والتيتانوس والسعال الديكي (جرعة اللقاح الثلاثي DTP1 وDTP3) عند الأطفال الرضع منخفضي الوزن عند الولادة في غانا، والمحددات المرتبطة بذلك.

**الطريقة:**

استخدمنا البيانات المستمدة من تجربة فيتامين ألف التي تستند إلى قطاعات سكانية معينة والتي تم إجراؤها في الفترة من 2010 إلى 2013 على 22,955 رضيع تم تسجيله. أجرينا قياسًا لمعدل تلقي اللقاح والخصائص المتعلقة بالأمهات والأطفال الرضع، كما أجرينا مقارنة بين ثلاث شرائح من الأطفال الرضع منخفضي الوزن عند الولادة (2.0 – 2.4 كلغ؛ و1.5 – 1.9 كلغ؛ والأقل من 1.5 كلغ) من جانب والأطفال الرضع ممن يبلغ وزنهم 2.5 كلغ أو يزيد عن ذلك من جانب آخر. وتم استخدام نموذج التحوف لبواسون لاحتساب نسب معدل تلقي اللقاح بشأن جرعة اللقاح الثلاثي DTP1 عند بلوغ 10 و14 و18 أسبوعًا بعد الولادة، وبشأن جرعة اللقاح الثلاثي DTP3 عند بلوغ 18 و22 و24 أسبوعًا (بما يساوي 1 و2 و3 أشهر بعد مرور 6 و14 أسبوعًا بعد حلول تواريخ وجوب تلقي اللقاحات المذكورة على التوالي).

**النتائج:**

كان معدل تلقي جرعة اللقاح الثلاثي DTP1 عند الأطفال الرضع منخفضي الوزن عند الولادة (العدد = 3382) أقل بنسبة تقرب من ‏40% مقارنةً بالأطفال الرضع ممن لا يعانون من انخفاض الوزن عند الولادة (العدد = 18,979)، وذلك في عمر 10 أسابيع (بنسبة المعدل المصححة، aRR: 0.58؛ ونسبة أرجحية مقدارها ‏95‏%: 0.43 – 0.77) وعند بلوغ 18 أسبوعًا (aRR: 0.63؛ ونسبة أرجحية مقدارها ‏95‏%: 0.50–0.80). وكانت معدلات تلقي اللقاح عند الأطفال الرضع ممن يبلغ وزنهم 1.5 - 1.9 (العدد = 386) أقل بنسبة تقرب من ‏25% مقارنةً بالأطفال الرضع ممن يبلغ وزنهم 2.5 كلغ أو أكثر في الأوقات المذكورة. وقد لوحظت نتائج مشابهة لذلك فيما يتعلق بجرعة اللقاح الثلاثي DTP3. وكان ثمة ارتباط بين صغر سن الأمهات، وقصر مدة تعليمهن، وبعد المسافة اللازمة للوصول إلى أقرب منشأة صحية من جانب وانخفاض معدلات تلقي جرعة اللقاح الثلاثي DTP1 وDTP3 من جانب آخر.

**الاستنتاج:**

يمثل الأطفال الرضع منخفضو الوزن عند الولادة مجموعة معرضة لدرجة عالية من الخطر فيما يتعلق بتأخر تلقي اللقاحات اللازمة في غانا. وتقتضي الحاجة بذل الجهود لتعزيز تلقي هؤلاء الأطفال الرضع للقاحات، إلى جانب إجراء المزيد من البحوث لإدراك أسباب التأخر في ذلك.

## 摘要

**目的:**

旨在调查加纳低出生体重婴儿在进行第一次和第三次白喉、破伤风、百日咳（DTP1 和 DTP3）疫苗接种时的延误情况和相关因素。

**方法:**

我们所采用的数据来源于 2010-2013 年间在 22 955 名接受调查的婴儿中开展的基于群体的大规模维生素 A 试验。 我们衡量了疫苗接种率和母亲及婴儿特征，并将三类低出生体重婴儿（2.0–2.4 公斤；1.5–1.9 公斤；和不足 1.5 公斤）与出生体重大于或等于  2.5 公斤的婴儿相比较，并在出生 10、14 和 18 周后使用泊松回归分析计算 DTP1 疫苗接种率，在 18、22 和 24 周后计算 DTP3 接种率【相当于两个接种截止日期（即 6 周和 14 周）后的第 1、2 和第 3 个月】。

**结果:**

低出生体重婴儿 (*n* = 3382) 在第 10 周时的 DTP1 接种率大约比非低出生体重婴儿 (*n* = 18 979) 低 40% （调整后的优势比，aRR: 0.58; 95% CI: 0.43–0.77），第 18 周 (aRR: 0.63; 95% CI: 0.50–0.80)。 在所取时间节点，体重在 1.5–1.9 公斤的婴儿 (*n* = 386) 接种率大约比体重大于或等于  2.5 公斤的婴儿低 25%。 DTP3 的观察结果相似。 较低的 DTP1 和 DTP3 接种率与孕产年龄较低、受教育程度较低和距离最近的医疗机构较远有关。

**结论:**

低出生体重儿童是加纳接种延迟的高危人群。 努力提高这些婴儿的免疫接种率是必要的，同时开展进一步调查，以了解延误原因。

## Резюме

**Цель:**

Изучить несоблюдение сроков первой и третьей вакцинации против дифтерии, коклюша и столбняка (АКДС1 и АКДС3) грудных детей со сниженной массой тела при рождении в Гане и связанные с этим определяющие факторы.

**Методы:**

Были использованы данные, полученные в результате обширного популяционного исследования приема витамина A, которое проводилось в 2010–2013 гг. при участии 22 955 грудных детей. Был определен охват вакцинацией матерей и грудных детей, были выявлены их характеристики, и были сопоставлены три категории грудных детей со сниженной (≥2,5 кг) массой тела при рождении: 2,0–2,4; 1,5–1,9 и <1,5 кг. С помощью регрессии Пуассона были подсчитаны отношения рисков вакцинации для вакцинации АКДС1, проведенной на 10, 14 и 18-й неделе после рождения, и для вакцинации АКДС3, проведенной на 18, 22 и 24-й неделе (что соответствует задержке на 1, 2 и 3 месяца по сравнению с установленными сроками такой вакцинации, проводимой на 6-й и 14-й неделях).

**Результаты:**

Для младенцев, имевших сниженную массу тела при рождении (n = 3382), охват вакцинацией АКДС1 был приблизительно на 40% ниже по сравнению с остальными младенцами (n = 18 979) в возрасте 10 недель (стандартизированное отношение рисков, СОР: 0,58; 95%-й ДИ: 0,43–0,77) и в возрасте 18 недель (СОР: 0,63; 95%-й ДИ: 0,50–0,80). Охват вакцинацией для младенцев с массой тела 1,5–1,9 кг (n = 386) был приблизительно на 25% ниже, чем для младенцев с массой тела ≥2,5 кг на тот же момент времени. Такие же результаты были получены для АКДС3. Более молодой возраст и меньший уровень образования матери, а также большее расстояние до ближайшего медицинского учреждения коррелировали с меньшим охватом вакцинациями АКДС1 и АКДС3.

**Вывод:**

Грудные дети со сниженной массой тела при рождении входят в группу высокого риска несоблюдения сроков вакцинации в Гане. Требуется предпринять меры для повышения охвата вакцинацией этих грудных детей, а также провести дополнительные исследования для понимания причин задержек.

## Introduction

Approximately 14% of infants born in low- and middle-income countries have a low birth weight (weighing < 2.50 kg at birth).^1^ It has been reported that in high-income settings, low-birth-weight infants have an increased risk of vaccine-preventable diseases, such as pertussis,^2^ invasive pneumococcal disease^3^^–^^5^ and *Haemophilus influenzae* type b (Hib).^6^ However, it is not known whether such risk exists in low-income settings. Timely vaccination of low-birth-weight infants, including booster doses, is important because these infants have lower passive immunity before vaccination^7^ and may respond sub-optimally to primary vaccination.^8^ Vaccination has similar efficacy and safety in low-birth-weight infants compared with non-low-birth-weight infants,^8^ and therefore vaccination is recommended at the same chronological age as other infants.^9^

Studies from high-income settings indicate that low-birth-weight infants are vaccinated later than non-low-birth-weight infants.^10^^,^^11^ Regardless of whether they are at increased risk, delayed vaccination of low-birth-weight infants prolongs their risk period for contracting vaccine-preventable diseases, especially Hib and *Streptococcus pneumoniae*,^3^^,^^12^ which are most prevalent in the first few months of life. Studies of the effect of low birth weight on timely vaccination in low-income settings, however, are lacking.

We aimed to measure the timing of vaccination of low-birth-weight infants compared with non-low-birth-weight infants by analysing data from a population-based, prospective cohort study in Ghana. Our primary objectives were to assess whether low birth weight is a determinant of delayed first and third dose diphtheria–tetanus–pertussis (DTP1 and DTP3) vaccination; and whether maternal education or socioeconomic status modified the association between birth weight and vaccination with DTP1 and DTP3. As a secondary objective, we aimed to quantify other determinants of delayed DTP1 and DTP3 vaccination.

## Methods

### Study design and setting

We studied a cohort of infants nested within a large randomized, double-blind, placebo-controlled trial of neonatal vitamin A supplementation conducted in Ghana between August 2010 and February 2013.^13^ The trial was conducted at the Kintampo Health Research Centre in Kintampo, Ghana. The trial procedures and study area have been described elsewhere.^14^

Ethics approval for the study was granted by the ethics committees of the World Health Organization (WHO), the London School of Hygiene & Tropical Medicine and the Kintampo Health Research Centre.

DTP vaccination in Ghana is recommended at 6, 10 and 14 weeks of age. Children are vaccinated at health facilities, community health planning system compounds or mobile outreach clinics. For each administered vaccine, the date and place of administration and vaccine batch number are usually documented in the child health record book. These may also be documented on a vaccination card or in the mother’s antenatal card. Infants who have never attended a child health clinic may not have a written record.

### Enrolment and data collection

Trained fieldworkers enrolled all consenting women aged 15–49 years residing in the study area into a reproductive surveillance system to document pregnancies and deliveries. All infants born in the study area were assessed for eligibility (eligible infants were aged ≤ 3 days at screening, could suck or feed and were staying in the study area for 6 months after enrolment) and mothers were asked for informed written consent for enrolment in the trial. Infants were weighed using calibrated electronic (38%; 8723 of enrolled infants) or spring (62%; 14 232) scales, to record birth weights to the nearest 0.1 kg (electronic scales) or 0.2 kg (spring scales). Only five (0.2%) infants were weighed later than 72 hours after delivery. The fieldworkers collected data on infant (sex and multiple delivery), maternal (age, education, occupation, and illness before delivery) and household characteristics (ethnicity, religion, socioeconomic status, distance to health facility and number of children in household).

The enrolled infants were visited monthly for the first year of life to collect data on the types and dates of vaccines given. We looked for written documentation of vaccines from all possible sources, including the child health record book, the mother’s antenatal card and vaccination cards. The infant’s caregiver (usually the mother) was also asked to recall what vaccines had been given. We also collected data on the infant’s vital status and on illnesses since the previous visit.

Follow-up started at birth. It ended at the vaccination date for vaccinated infants, and the end of the risk period for unvaccinated infants not lost to follow-up. For those lost to follow-up before the end of the risk period, follow-up ended on the last date the written record was viewed, for unvaccinated infants whose record was viewed; or on the last date the infant was seen, for unvaccinated infants whose record was never viewed; or on the date of death, for unvaccinated infants who died before the end of the risk period and whose record was viewed after their death.

For the analyses we included all infants from the trial with known vaccination status and dates and with complete data on covariates. We excluded infants who were lost to follow-up or died before the vaccination due date.

#### Definitions

We classified infants’ vaccination status as follows: (i) vaccinated, date known (written record had a plausible vaccination date); (ii) vaccinated, date unknown (record had clearly documented vaccination but with the date missing, illegible or implausible); (iii) unvaccinated (record was seen but had no documented vaccination date or any evidence of vaccination; or record was never seen and mother consistently reported infant had never been vaccinated); or (iv) vaccination status unknown (mother reported that infant had been vaccinated but did not specify the vaccine; or infant was never seen in follow-up).

We categorized birth weight into four standard categories: ≥ 2.5 kg (i.e. non-low birth weight); 2.0–2.4 kg; 1.5–1.9 kg; < 1.5 kg.^1^^,^^15^

#### Outcome measures

The study outcomes were delayed receipt of DTP1 and DTP3. There is no standard approach to the assessment of delayed vaccination and several definitions based on predefined cut-offs have been described.^16^^–^^18^ To assess how the effect of birth weight may vary over time, we defined risk periods for delayed vaccination up to 4, 8 and 12 weeks after the vaccination due date. For DTP1 we therefore analysed vaccination rates from birth up to 10, 14 and 18 weeks of age. For DTP3 we analysed vaccination from birth up to 18, 22 and 26 weeks of age.

### Data analysis

The data were double-entered and processed at the Kintampo Health Research Centre using the SQL Server 2008 data management system (Microsoft Corp., Redmond, USA). Inconsistencies and errors in the vaccination dates were corrected, with senior fieldworkers visiting mothers to review the written record and verify the dates if necessary.

**Fig. 1:**
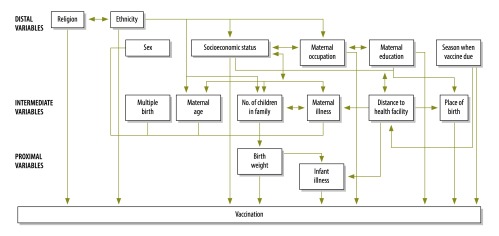
Hierarchical framework of determinants of infant vaccination in the prospective cohort study in rural Ghana, 2010–2013

Note: The effects of distal variables were hypothesized to be mediated by intermediate and proximal variables, and intermediate variables by the proximal variables. Among the proximal variables, illness was hypothesized to mediate the effect of birth weight. Mediation is indicated by arrows linking the variables across different levels.

All analyses were conducted using the Stata package version 13.1 (StataCorp, College Station, USA). We generated Kaplan–Meier curves of time to vaccination in low-birth-weight compared with non-low-birth-weight infants in the first year of life for DTP1 and DTP3. Vaccination rate ratios, adjusted for a priori selected factors, were obtained for each risk period using multivariable Poisson regression, informed by a hierarchical framework of the recognized determinants of vaccination (Fig. 1).^12^^,^^16^^–^^18^ The initial model included distal determinants of vaccination, then intermediate determinants were added, followed by birth weight and, finally, infant illness at the time the vaccine was due (as this was considered to be a possible mediator of the association between birth weight and vaccination).^19^ We assessed the statistical association between vaccination and each explanatory variable using likelihood ratio tests and 95% confidence intervals (CI). We also investigated whether the association between birth weight and vaccination varied by maternal education or socioeconomic status by testing the interaction of birth weight with these variables.

Two sets of sensitivity analyses were undertaken. First, to assess whether delayed DTP3 vaccination simply reflected delayed DTP1 vaccination, we repeated the DTP3 analyses, starting follow-up at receipt of DTP1 vaccination and ending 12 weeks after receipt of DTP1. Second, to examine the effect of possible misclassification of vaccine status for infants categorized as never vaccinated but whose written record was never viewed, we excluded these infants and repeated the analyses of DTP1 vaccination up to 18 weeks from birth and DTP3 vaccination up to 26 weeks.

## Results

**Fig. 2:**
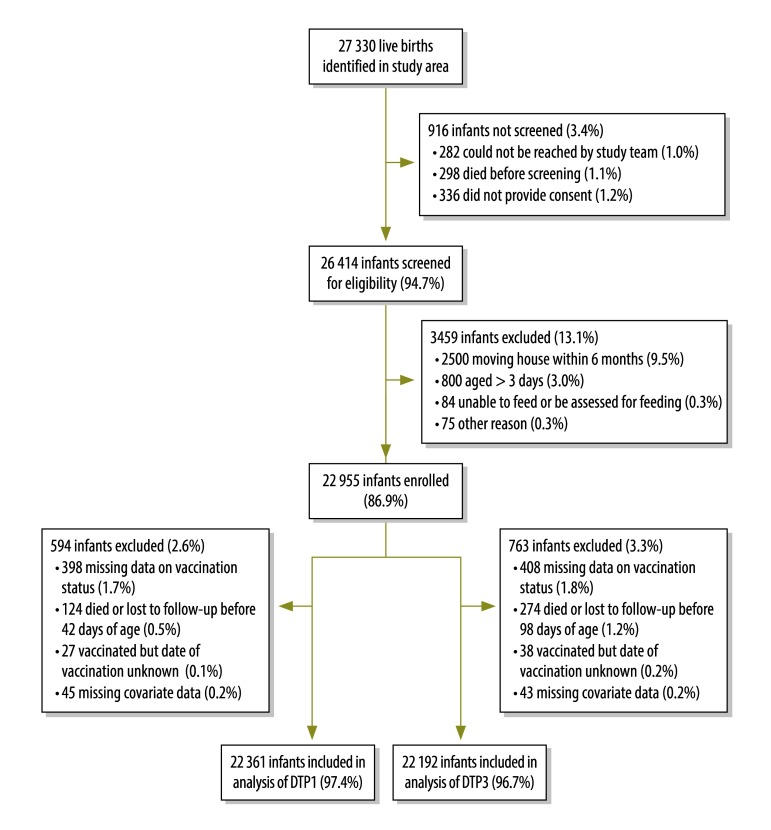
Identification, recruitment and inclusion of participants in the prospective cohort study on infant vaccination in rural Ghana, 2010–2013

DTP1: first dose of diphtheria–tetanus–pertussis vaccine; DTP3: third dose of diphtheria–tetanus–pertussis vaccine.

Note: Overall and disaggregated percentages may not agree due to rounding.

Of 27 330 live births identified in the study area, 26 414 infants were screened for eligibility for the trial and 22 955 were enrolled (Fig. 2); 22 361 (97.4%) and 22 192 (96.7%) infants were included in the analysis of DTP1 and DTP3 respectively. Low-birth-weight infants were more likely to be excluded from our analysis, as were those with illness reported around the vaccination due date, those from multiple births and those born to mothers of lower socioeconomic status, of non-Akan ethnicity, with lower education, with lower employment grades or living more than 5.0 km from a health facility (Table 1). Of the infants included in the DTP1 analysis, 18 979 (84.9%) were normal birth weight and 3382 (15.1%) were low birth weight: 2916 (13.0%) weighed 2.0–2.4 kg, 386 (1.7%) 1.5–1.9 kg and 80 (0.4%) < 1.5 kg. The birth weight distribution was the same for infants in the DTP3 analysis: 18 850 (84.9%) weighed ≥ 2.5 kg, 2886 (13.0%) 2.0–2.4 kg, 378 (1.7%) 1.5–1.9 kg and 78 (0.4%) < 1.5 kg (Table 1).

**Table 1:** Characteristics of infants vaccinated with first and third doses of diphtheria–tetanus–pertussis vaccine in the prospective cohort study in rural Ghana, 2010–2013

Characteristic	No. (%)				
	DTP1			DTP3	
	Included infants (*n* = 22 361)	Excluded infants (*n* = 594)		Included infants (*n* = 22 192)	Excluded infants (*n* = 763)
**Distal determinants**					
Religion of head of household					
Christian	15 616 (69.8)	363 (61.1)		15 497 (69.8)	482 (63.2)
Muslim	5 333 (23.8)	178 (30.0)		5 294 (23.9)	217 (28.4)
None/traditional/other	1 412 (6.3)	53 (8.9)		1401 (6.3)	64 (8.4)
Ethnicity of household					
Akan	10 470 (46.8)	223 (37.5)		10 410 (46.9)	283 (37.1)
Other	11 891 (53.2)	371 (62.5)		11 782 (53.1)	480 (62.9)
Socioeconomic status^a^					
1 (poorest)	4 356 (19.5)	155 (26.1)		4 299 (19.4)	212 (27.8)
2	4 407 (19.7)	143 (24.1)		4 363 (19.7)	187 (24.5)
3	4 469 (20.0)	113 (19.0)		4 440 (20.0)	142 (18.6)
4	4 544 (20.3)	100 (16.8)		4 523 (20.4)	121 (15.9)
5 (richest)	4 585 (20.5)	83 (14.0)		4 567 (20.6)	101 (13.2)
Maternal occupation					
Government/private/other	1 200 (5.4)	25 (4.2)		1 199 (5.4)	26 (3.4)
Self-employed	8 752 (39.1)	194 (32.7)		8 716 (39.3)	230 (30.1)
Farming	6 472 (28.9)	199 (33.5)		6 411 (28.9)	260 (34.1)
Not working	5 937 (26.6)	176 (29.6)		5 866 (26.4)	247 (32.4)
Maternal education					
None	6 913 (30.9)	214 (36.0)		6 845 (30.8)	282 (37.0)
Primary school	4 115 (18.4)	121 (20.4)		4 081 (18.4)	155 (20.3)
Secondary/tertiary	11 333 (50.7)	245 (41.2)		11 266 (50.8)	312 (40.9)
Missing values	0 (0.0)	14 (2.4)		0 (0.0)	14 (1.8)
Season when vaccine due: wet	14 176 (63.4)	382 (64.3)		10 406 (46.9)	347 (45.5)
Infant sex: female	11 025 (49.3)	281 (47.3)		10 938 (49.3)	368 (48.2)
**Intermediate determinants**					
Maternal age (years)					
< 20	2 550 (11.4)	95 (16.0)		2 514 (11.3)	131 (17.2)
20–24	5 714 (25.6)	173 (29.1)		5 657 (25.5)	230 (30.1)
25–29	6 017 (26.9)	137 (23.1)		5 986 (27.0)	168 (22.0)
30–34	4 522 (20.2)	95 (16.0)		4 497 (20.3)	120 (15.7)
≥ 35	3 558 (15.9)	64 (10.8)		3 538 (15.9)	84 (11.0)
Missing value	0 (0.0)	30 (5.1)		0 (0.0)	30 (3.9)
No. of children in family					
0–1	6 516 (29.1)	216 (36.4)		6 450 (29.1)	282 (37.0)
2–3	8 946 (40.0)	209 (35.2)		8 887 (40.0)	268 (35.1)
≥ 4	6 899 (30.9)	169 (28.5)		6 855 (30.9)	213 (27.9)
Maternal illness: yes	1 093 (4.9)	30 (5.1)		1 090 (4.9)	33 (4.3)
Distance from health facility (km)					
< 1.0	13 545 (60.6)	342 (57.6)		13 461 (60.7)	436 (57.1)
1.0–4.9	5 147 (23)	117 (19.7)		5 106 (23.0)	151 (19.8)
≥ 5.0	3 669 (16.4)	133 (22.4)		3 625 (16.3)	169 (22.1)
Missing value	0 (0.0)	2 (0.3)		0 (0.0)	7 (0.9)
Place of birth: health facility	17 155 (76.7)	426 (71.7)		17 047 (76.8)	534 (70.0)
Multiple birth	795 (3.6)	52 (8.8)		784 (3.5)	63 (8.3)
Birth weight (kg)					
≥ 2.5	18 979 (84.9)	382 (64.3)		18 850 (84.9)	511 (67.0)
2.0–2.4	2 916 (13.0)	115 (19.4)		2 886 (13.0)	145 (19.0)
1.5–1.9	386 (1.7)	58 (9.8)		378 (1.7)	66 (8.7)
< 1.5	80 (0.4)	37 (6.2)		78 (0.4)	39 (5.1)
Missing value	0 (0.0)	2 (0.3)		0 (0.0)	2 (0.3)
**Mediating variables**					
Infant illness: yes	2 748 (12.3)	155 (26.1)		3 429 (15.5)	277 (36.3)
Missing value	0 (0.0)	261 (43.9)		0 (0.0)	329 (43.1)

DTP1: first dose of diphtheria–tetanus–pertussis vaccine; DTP3: third dose of diphtheria–tetanus–pertussis vaccine.

^a^ Socioeconomic status was calculated by principal components analysis from an inventory of household assets.

### Delayed vaccination

#### Birth weight

Although uptake of vaccination was high (> 95%) for all infants by 1 year of age, low birth weight was associated with later vaccination for both DTP1 and DTP3. Median ages at DTP1 vaccination were 8 weeks (interquartile range, IQR: 6.7–9.6 weeks) for infants weighing ≥ 2.5 kg at birth; 8.3 weeks (IQR: 6.9–9.9) for those 2.0–2.4 kg; 8.4 weeks (IQR: 6.9–10.7) for those 1.5–1.9 kg and 9 weeks (IQR: 7.4–11.9) for those < 1.5 kg. For DTP3, the corresponding median ages at vaccination were 18.4 weeks (IQR: 16.3–22.1), 18.6 weeks (IQR: 16.6–22.3), 19.6 weeks (IQR: 16.6–23.3) and 20.4 weeks (IQR: 17.7–25.1), respectively.

**Fig. 3:**
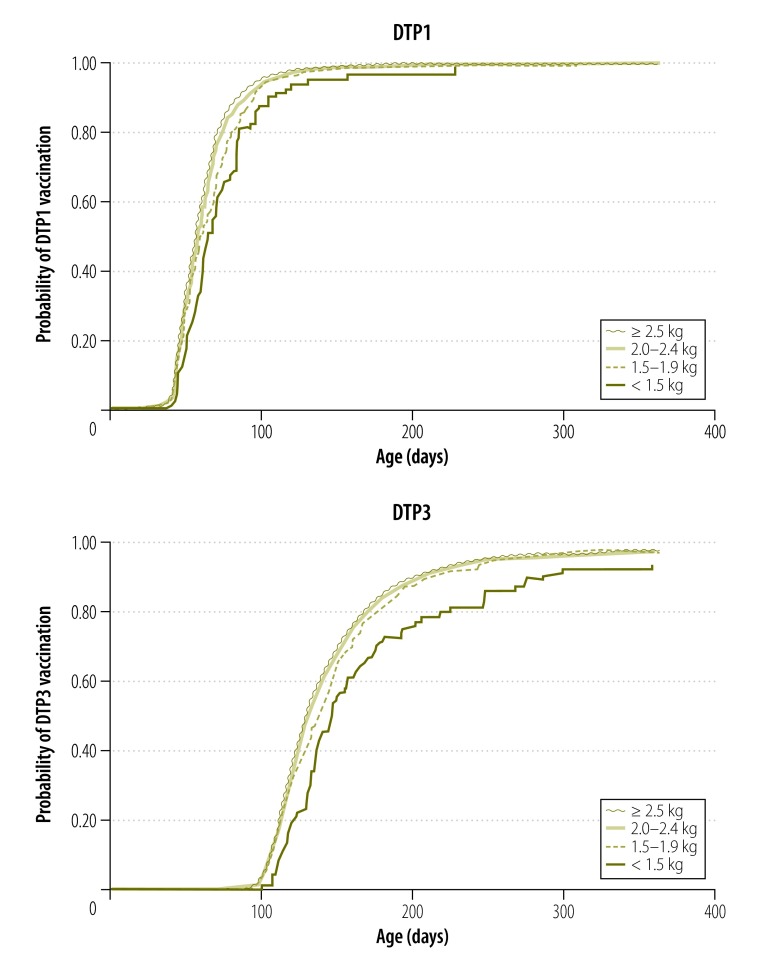
Time to vaccination with first dose and third dose of diphtheria–tetanus–pertussis vaccine, by birth weight in the prospective cohort study in rural Ghana, 2010–2013

DTP1: first dose of diphtheria–tetanus–pertussis vaccine; DTP3: third dose of diphtheria–tetanus–pertussis vaccine.

The Kaplan–Meier curves showed that DTP1 vaccination rates over the days since birth were also lower for infants weighing < 1.5 kg and those weighing 1.5–1.9 kg compared with those weighing ≥ 2.5 kg (Fig. 3**)**. After adjustment for other variables, there was evidence of progressively delayed vaccination with decreasing birth weight (*P-*value for trend < 0.0001). Infants weighing < 1.5 kg at birth had a DTP1 vaccination rate approximately 40% lower than non-low-birth-weight infants by the age of 10 weeks (adjusted rate ratio, aRR: 0.58; 95% CI: 0.43–0.77) and age 18 weeks (aRR: 0.63; 95% CI: 0.50–0.80). Infants weighing 1.5–1.9 kg had vaccination rates approximately 25% lower than non-low-birth-weight infants at these time points (aRR: 0.71; 95% CI: 0.62–0.81 and aRR: 0.76; 95% CI: 0.69–0.85, respectively; Table 2, available at: http://www.who.int/bulletin/volumes/94/5/15-159699).

**Table 2:** Birth weight as a determinant of vaccination of infants with first and third doses of diphtheria–tetanus–pertussis vaccine at various ages, rural Ghana, 2010–2013

Vaccine and age	No. of vaccinations/ no. of person-days of follow-up	Vaccination rate per 100 days of follow-up (95%CI)	Unadjusted RR			aRR^a^			aRR, additionally adjusted for infant illness	
			RR (95% CI)	*P*		aRR (95% CI)	*P*^b^		aRR (95% CI)	*P*
**DTP1 at age 10 weeks**										
≥ 2.5 kg	14 759/1 065 163	1.39 (1.36–1.41)	Ref	< 0.0001		Ref	< 0.0001		Ref	< 0.0001
2.0–2.4 kg	2 185/166 348	1.31 (1.26–1.37)	0.95 (0.91–0.99)			0.93 (0.89–0.97)			0.93 (0.89–0.97)	
1.5–1.9 kg	243/22 400	1.08 (0.96–1.23)	0.78 (0.69–0.89)			0.71 (0.62–0.81)			0.71 (0.63–0.81)	
< 1.5 kg	45/4 886	0.92 (0.69–1.23)	0.66 (0.50–0.89)			0.58 (0.43–0.77)			0.58 (0.43–0.78)	
**DTP1 at age 14 weeks**										
≥ 2.5 kg	17 789/1 126 945	1.58 (1.56–1.60)	Ref	0.0064		Ref	< 0.0001		Ref	< 0.0001
2.0–2.4 kg	2680/177 815	1.51 (1.45–1.57)	0.95 (0.92–0.99)			0.92 (0.88–0.96)			0.92 (0.88–0.96)	
1.5–1.9 kg	347/24 482	1.42 (1.28–1.57)	0.90 (0.81–1.00)			0.77 (0.69–0.86)			0.77 (0.69–0.86)	
< 1.5 kg	69/5 497	1.26 (0.99–1.59)	0.80 (0.63–1.01)			0.62 (0.49–0.79)			0.63 (0.49–0.80)	
**DTP1 at age 18 weeks**										
≥ 2.5 kg	18 427/1 145 653	1.61 (1.59–1.63)	Ref	0.0205		Ref	< 0.0001		Ref	< 0.0001
2.0–2.4 kg	2 810/181 294	1.55 (1.49–1.61)	0.96 (0.93–1.00)			0.92 (0.89–0.96)			0.92 (0.89–0.96)	
1.5–1.9 kg	364/25 020	1.45 (1.31–1.61)	0.90 (0.82–1.00)			0.76 (0.69–0.85)			0.76 (0.69–0.85)	
< 1.5 kg	75/5 708	1.31 (1.05–1.65)	0.82 (0.65–1.02)			0.63 (0.50–0.80)			0.63 (0.50–0.79)	
**DTP3 at age 18 weeks**										
≥ 2.5 kg	8 007/2 240 325	0.36 (0.35–0.37)	Ref	0.0005		Ref	< 0.0001		Ref	< 0.0001
2.0–2.4 kg	1 168/344 907	0.34 (0.32–0.36)	0.95 (0.89–1.01)			0.93 (0.88–0.99)			0.93 (0.88–0.99)	
1.5–1.9 kg	132/45 006	0.29 (0.25–0.35)	0.82 (0.69–0.97)			0.78 (0.66–0.93)			0.78 (0.66–0.93)	
< 1.5 kg	17/9 381	0.18 (0.11–0.29)	0.51 (0.32–0.82)			0.46 (0.29–0.75)			0.46 (0.29–0.75)	
**DTP3 at age 22 weeks**										
≥ 2.5 kg	13 238/245 2 731	0.54 (0.53–0.55)	Ref	0.0246		Ref	< 0.0001		Ref	0.0001
2.0–2.4 kg	1 992/378 547	0.53 (0.50–0.55)	0.97 (0.93–1.02)			0.96 (0.91–1.01)			0.96 (0.91–1.01)	
1.5–1.9 kg	239/49 991	0.48 (0.42–0.54)	0.89 (0.78–1.01)			0.80 (0.70–0.92)			0.80 (0.70–0.92)	
< 1.5 kg	41/10 583	0.39 (0.29–0.53)	0.72 (0.53–0.98)			0.61 (0.45–0.83)			0.61 (0.45–0.83)	
**DTP3 at age 26 weeks**										
≥ 2.5 kg	15 694/2 559 854	0.61 (0.60–0.62)	Ref	0.0334		Ref	< 0.0001		Ref	< 0.0001
2.0–2.4 kg	2 360/395 994	0.60 (0.57–0.62)	0.97 (0.93–1.01)			0.95 (0.91–1.00)			0.95 (0.91–1.00)	
1.5–1.9 kg	296/52 518	0.56 (0.50–0.63)	0.92 (0.82–1.03)			0.82 (0.73–0.92)			0.82 (0.73–0.93)	
< 1.5 kg	51/11 303	0.45 (0.34–0.59)	0.74 (0.56–0.97)			0.60 (0.45–0.79)			0.60 (0.46–0.79)	
**DTP3 within 12 weeks of DTP1**										
≥ 2.5 kg	11 090/375 642	2.95 (2.90–3.01)	Ref	< 0.0001		Ref	0.0026		Ref	0.0069
2.0–2.4 kg	1 664/60 515	2.75 (2.62–2.89)	0.93 (0.88–0.98)			0.93 (0.88–0.98)			0.93 (0.88–0.98)	
1.5–1.9 kg	202/7 548	2.68 (2.33–3.07)	0.91 (0.79–1.04)			0.98 (0.85–1.13)			0.98 (0.85–1.12)	
< 1.5 kg	32/2 330	1.37 (0.97–1.94)	0.47 (0.33–0.66)			0.65 (0.46–0.92)			0.65 (0.46–0.93)	

aRR: adjusted rate ratio; CI: confidence interval; DTP1: first dose of diphtheria–tetanus–pertussis vaccine; DTP3: third dose of diphtheria–tetanus–pertussis vaccine; Ref: reference group; RR: rate ratio.

^a^ Adjusted for ethnicity, religion, socioeconomic status, maternal occupation, maternal education, season when vaccine due, infant sex, maternal age, family size, maternal illness in year before delivery, distance from health facility, place of delivery, multiple birth and age-band of infant.

^b^ *P*-value for linear trend.

Similar results were observed for DTP3 (Fig. 3). The findings were also similar for DTP1 and DTP3 vaccination at 8 weeks after the due date (Table 2).

Adjusting for illness had little effect on the magnitude of the association between birth weight and vaccination for both DTP1 and DTP3 (Table 2).

#### Other variables

Younger maternal age, lower educational attainment, and longer distance to the nearest health facility were associated with moderate reductions in the DTP1 and DTP3 vaccination rates of approximately 10–20% at ages 10 and 18 weeks, whereas higher employment grade was associated with moderate increased vaccination rates at these ages (Table 3, available at: http://www.who.int/bulletin/volumes/94/5/15-159699). In the final model (after adjusting for potential mediating variables) low socioeconomic status of mothers was associated with a 15% increased DTP3 vaccination rate at 18 weeks, whereas no association with DTP1 vaccination was observed. Muslim religion and larger family size were associated with > 10% reduction in DTP3 vaccination rates but had no, or only a small, association with DTP1 vaccination rates. None of the other variables measured had notable associations with DTP1 or DTP3 vaccination rates at any ages.

**Table 3:** Determinants of delayed vaccination with first dose diphtheria–tetanus–pertussis vaccine at age 10 weeks and third dose diphtheria–tetanus–pertussis vaccine at age 18 weeks for infants, rural Ghana, 2010–2013

Determinants	DTP1 at age 10 weeks						DTP3 at age 18 weeks			
	Unadjusted RR			aRR^a^			Unadjusted RR		aRR^a^	
	RR (95% CI)		*P*	aRR (95% CI)	*P*		RR (95% CI)	*P*	aRR (95% CI)	*P*
**Distal determinants**										
Religion of head of household										
Christian		Ref	0.0002	Ref	0.0382		Ref	< 0.0001	Ref	< 0.0001
Muslim		0.93 (0.90–0.97)		0.95 (0.91–0.99)			0.77 (0.73–0.81)		0.81 (0.77–0.86)	
None/traditional/other		0.94 (0.88–1.00)		0.99 (0.93–1.06)			0.93 (0.86–1.01)		1.01 (0.93–1.10)	
Ethnicity of household										
Akan		Ref	< 0.0001	Ref	0.0728		Ref	< 0.0001	Ref	0.3484
Other		0.94 (0.91–0.96)		1.04 (1.00–1.08)			0.85 (0.82–0.89)		1.03 (0.97–1.08)	
Socioeconomic status										
1 (poorest)		0.84 (0.80–0.88)	< 0.0001	0.96 (0.90–1.02)	0.5398		0.87 (0.81–0.93)	0.0001	1.13 (1.04–1.23)	0.0010
2		0.91 (0.87–0.95)		1.00 (0.94–1.05)			0.95 (0.89–1.02)		1.15 (1.07–1.24)	
3		0.93 (0.89–0.97)		0.98 (0.93–1.03)			1.00 (0.94–1.06)		1.13 (1.06–1.21)	
4		0.95 (0.91–1.00)		0.98 (0.94–1.03)			0.97 (0.91–1.03)		1.05 (0.99–1.12)	
5 (richest)		Ref		Ref			Ref		Ref	
Maternal occupation										
Government/private/other		1.08 (1.01–1.16)	< 0.0001	1.09 (1.02–1.17)	0.0031		1.16 (1.06–1.27)	0.0013	1.11 (1.01–1.21)	0.0394
Self-employed		Ref		Ref			Ref		Ref	
Farming		0.90 (0.87–0.94)		0.95 (0.91–0.99)			0.96 (0.92–1.01)		1.07 (1.00–1.13)	
Not working		0.95 (0.92–0.99)		0.99 (0.95–1.03)			0.99 (0.94–1.04)		1.05 (0.99–1.11)	
Maternal education										
None		0.88 (0.85–0.91)	< 0.0001	0.88 (0.84–0.92)	< 0.0001		0.77 (0.73–0.81)	< 0.0001	0.77 (0.72–0.81)	< 0.0001
Primary school		0.93 (0.89–0.96)		0.93 (0.89–0.97)			0.84 (0.80–0.89)		0.85 (0.80–0.90)	
Secondary/tertiary		Ref		Ref			Ref		Ref	
Season when vaccine due										
Wet		Ref	0.2971	Ref	0.1817		Ref	0.0480	Ref	0.0511
Dry		0.98 (0.95–1.01)		0.98 (0.95–1.01)			0.96 (0.92–1.00)		0.96 (0.92–1.00)	
Infant sex										
Male		Ref	0.5352	Ref	0.2206		Ref	0.2602	Ref	0.1165
Female		1.01 (0.98–1.04)		1.02 (0.99–1.05)			1.02 (0.98–1.07)		1.03 (0.99–1.08)	
**Intermediate determinants**										
Maternal age, years										
< 20		0.90 (0.86–0.95)	0.0053	0.84 (0.78–0.89)	< 0.0001		0.87 (0.80–0.93)	0.0030	0.77 (0.70–0.84)	< 0.0001
20–24		0.97 (0.93–1.01)		0.93 (0.89–0.98)			0.97 (0.92–1.03)		0.93 (0.88–0.99)	
25–29		Ref		Ref			Ref		Ref	
30–34		0.98 (0.94–1.02)		1.03 (0.98–1.07)			0.99 (0.93–1.05)		1.06 (0.99–1.13)	
≥ 35		0.96 (0.91–1.00)		1.02 (0.97–1.08)			0.96 (0.90–1.02)		1.06 (0.99–1.14)	
No. of children in family										
0–1		1.00 (0.96–1.03)	0.0103	1.05 (1.00–1.10)	0.0066		1.00 (0.95–1.05)	0.0039	1.07 (1.01–1.13)	0.0004
2–3		Ref		Ref			Ref		Ref	
≥ 4		0.95 (0.92–0.98)		0.95 (0.91–1.00)			0.93 (0.88–0.97)		0.92 (0.86–0.97)	
Maternal illness										
No		Ref	0.4939	Ref	0.1319		Ref	0.9296	Ref	0.7234
Yes		1.02 (0.96–1.10)		1.05 (0.98–1.13)			1.00 (0.91–1.09)		1.02 (0.93–1.12)	
Distance from health facility (km)										
< 1.0		Ref	< 0.0001	Ref	< 0.0001		Ref	< 0.0001	Ref	< 0.0001
1.0–4.9		0.95 (0.92–0.99)		0.94 (0.90–0.97)			0.93 (0.88–0.98)		0.90 (0.86–0.95)	
≥ 5.0		0.85 (0.81–0.89)		0.86 (0.82–0.91)			0.78 (0.73–0.83)		0.79 (0.74–0.84)	
Place of birth										
Health facility		Ref	< 0.0001	Ref	0.0065		Ref	< 0.0001	Ref	0.0281
Non-facility		0.89 (0.86–0.92)		0.94 (0.91–0.98)			0.86 (0.82–0.91)		0.94 (0.89–0.99)	
Multiple birth										
No		Ref	0.1420	Ref	0.4172		Ref	0.8390	Ref	0.1225
Yes		0.94 (0.87–1.02)		1.04 (0.95–1.13)			0.99 (0.89–1.10)		1.10 (0.98–1.24)	
**Mediating variables**										
Infant illness										
No		Ref	0.0507	Ref	0.0949		Ref	0.1540	Ref	0.2632
Yes		0.96 (0.91–1.00)		0.95 (0.91–1.00)			0.96 (0.91–1.02)		1.02 (0.96–1.08)	

aRR: adjusted rate ratio; CI: confidence interval; DTP1: first dose of diphtheria–tetanus–pertussis vaccine; DTP3: third dose of diphtheria–tetanus–pertussis vaccine; Ref: reference group; RR: rate ratio.

^a^ Also adjusted for infant age-band.

### Sensitivity analyses

Adjusting for late vaccination with DTP1 decreased the effect size for the association between birth weight and the rate of DTP3 vaccination for infants weighing 1.5–1.9 kg (12 weeks after DTP1 aRR: 0.98; 95% CI: 0.85–1.13) compared with an aRR of 0.82 (95% CI: 0.73–0.92) at 12 weeks after the DTP3 due date, but the effect size for infants weighing < 1.5 kg was largely unchanged (Table 2).

Excluding unvaccinated infants whose written record was never seen had little impact on the effect size of the explanatory variables for DTP1 or DTP3.

### Modifying factors

When we looked at other factors that might modify the association between birth weight and delayed vaccination there was no evidence that the effect of birth weight on vaccination with DTP1 or DTP3 varied by socioeconomic status (*P*-values for interaction all > 0.4), or that the rate of vaccination with DTP1 varied by maternal education, when measured at age 10 weeks (*P* = 0.3338) or age 18 weeks (*P* = 0.2675). However, for DTP3 vaccination there was some evidence that the effect of birth weight on the vaccination rate at age 18 weeks (*P* = 0.0219) and age 26 weeks (*P* = 0.0813) varied with maternal education, with a more pronounced reduction in vaccination rate among smaller infants born to mothers with higher educational attainment (aRR for infants weighing < 1.50 kg at age 18 weeks: 0.37; 95% CI: 0.19–0.72; aRR at 26 weeks: 0.63; 95% CI: 0.50–0.80). When infants with delayed receipt of DTP1 were excluded from the analysis, this effect was no longer apparent.

## Discussion

The results of this study provide evidence that low-birth-weight infants in Ghana are vaccinated later than non-low-birth-weight infants. The effect persisted up to 12 weeks after the vaccination due date and was evident for both DTP1 and DTP3, even after controlling for other determinants of delayed vaccination.

The results are consistent with previous reports from high-income countries of delayed vaccination in low-birth-weight infants.^10^^,^^11^^,^^20^^–^^22^ In addition, a study of low-birth-weight infants in Guinea-Bissau, which did not look at timeliness, reported lower uptake of DTP1 at 8 weeks of age among smaller low-birth-weight infants compared with larger low-birth-weight infants.^23^ A North American study reported that both parents and vaccine-providers had erroneous beliefs that initiation of vaccination depended on the degree of prematurity and the infant’s weight.^22^ In addition, a review of 47 studies in the grey literature from low-income settings reported parental reluctance to bring sick, weak or malnourished children for vaccination for reasons of social stigma and fatalism; these have also been cited as reasons for non-vaccination by vaccine providers.^19^

Low-birth-weight infants in low-income settings are known to have higher rates of illness and death in the first year of life than non-low-birth-weight infants.^15^^,^^24^^–^^27^ Data from high-income settings indicate that they also have higher rates of illness from vaccine-preventable diseases.^2^^–^^4^^,^^6^ The risk and consequences of illness related to vaccine-preventable diseases in low-birth-weight infants in low-income settings is not known and may differ from those in high-income settings. Without this information it is difficult to fully understand the implications of delayed vaccination on clinical outcomes for these infants. However, we do know that delayed vaccination of these infants will prolong their risk period for contracting these diseases and may also reflect an underuse of health services by the caregivers of these infants. Given this increased risk of illness and death, it is essential that all opportunities for vaccination and health care for low-birth-weight infants be exploited.

We also identified several additional determinants of delayed vaccination – low maternal age and educational attainment and longer distance to the nearest health facility – that reflect persisting inequities in access to and uptake of vaccination in our study population. This is consistent with previous findings from the study area^16^ and the issue of inequities in coverage of vaccination have featured in global vaccine policy.^28^

The strengths of our study include the high quality population-based surveillance system and low loss to follow-up. Almost all infants were weighed within 72 hours of delivery by trained fieldworkers using calibrated scales, thus minimizing the likelihood of misclassification of infants by birth weight. Similarly, we collected high quality data on vaccination – from both written records and maternal recall – and we employed a rigorous approach to resolving inconsistencies in these data. Although recall data is used in the generation of routine vaccine uptake estimates,^29^ their validity may vary.^30^^,^^31^ The validity of our recall data was maximized by the continuous nature of the data collection in our study. Infants with recall data accounted for less than 0.6% of all infants included in the analyses and had little impact on our estimates. Furthermore, the inclusion of over 22 000 infants ensured that the study had sufficient power to show effects in small subgroups.

Aspects of this study that may have affected the generalizability of our findings are that our study sample may have experienced more timely vaccination compared with the general population. A higher proportion of low-birth-weight infants than non-low-birth-weight infants were excluded from our analyses, either because they did not meet the inclusion criteria for enrolment in the trial or because more of them were lost to follow-up or had missing data (including missing vaccination data) than non-low-birth-weight infants. Those excluded could have experienced greater delay in receiving their vaccines compared with the included low-birth-weight infants, possibly causing some underestimation of the association between low birth weight and timely vaccination in our population. As less than 5% of enrolled infants were excluded, this was unlikely to have changed the results appreciably and important delays in vaccination were still observed among low-birth-weight infants. Mothers of enrolled infants were asked about their infant’s vaccination status at monthly visits, possibly increasing their awareness of the need to vaccinate their infants. This increased awareness, however, would not have been differentially affected by birth weight and would lead to an overall underestimation of delayed vaccination.

Other limitations are that we did not have reliable data on gestational age and therefore we were not able to assess whether delayed vaccination was associated with prematurity or whether all low-birth-weight infants were affected regardless of gestational age. This study was also not designed to assess the association between delayed vaccination and clinical outcomes such as vaccine-preventable diseases or hospitalizations. Consequently, we do not know whether those infants who had delayed vaccination were more likely to contract vaccine-preventable diseases or to report elevated rates of illness or hospitalization. Eleven explanatory variables were included in our secondary analysis, thus increasing the potential for type 1 errors (finding statistically significant results by chance alone). Finally, we did not collect any qualitative data on the reasons for delayed vaccination of low-birth-weight infants in our study sample. This limits our interpretation of the findings. It may be that vaccination was delayed for reasons beyond the control of both the caregivers and the vaccine providers, such as lack of vaccines or staff, although it is reasonable to assume that these would not be distributed differently among low-birth-weight compared with non-low-birth-weight infants.

### Recommendations

Current global policy on vaccination advocates the identification of groups that are underserved by vaccination,^28^ yet data on uptake and timeliness of vaccination in low-birth-weight infants are not currently included in routine evaluations of vaccination programmes. These data are feasible to collect in low-income settings; doing this would contribute to a more comprehensive evaluation of the performance of vaccination programmes and would inform the development of strategies to improve uptake and timing of vaccination in all countries.^28^ Even though several organizations in high-income countries have made specific recommendations about vaccination of low-birth-weight infants,^9^^,^^32^ international guidelines are lacking.

Efforts to improve the vaccination of low-birth-weight infants, for example by education of caregivers and vaccine-providers, are warranted. Further research is needed in low-income countries to understand the reasons for delayed vaccination of low-birth-weight infants and to inform strategies to improve the timeliness of vaccination.
